# Surveillance and Molecular Characterization of Marek’s Disease Virus (MDV) Strains Circulating in Tanzania

**DOI:** 10.3390/v17050698

**Published:** 2025-05-13

**Authors:** Augustino Alfred Chengula, Herbertha Mpete, Ramadhani Juma Makasali

**Affiliations:** Department of Veterinary Microbiology, Parasitology and Biotechnology, Sokoine University of Agriculture, Morogoro P.O. Box 3019, Tanzania; herbertha.mpete@sua.ac.tz (H.M.); ramadhani.makasali@sua.ac.tz (R.J.M.)

**Keywords:** Marek’s disease, Marek’s disease virus, PCR, sequencing, oncogenic genes, chicken

## Abstract

Marek’s disease (MD) is a highly contagious and oncogenic viral disease of poultry, causing significant economic losses due to mortality and reduced performance. The rapid evolution of Marek’s disease virus (MDV) has been reported in poultry farms, often overcoming vaccination and leading to disease outbreaks. This study aimed to detect and molecularly characterize circulating MDV strains in Tanzania, with a focus on their genetic relationship with the vaccine strains currently in use (HVT and CVI988). Samples were collected from six livestock representative zones in Tanzania (Central, Eastern, Southern, Southern Highlands, Lake, and Northern Zone) and analyzed using polymerase chain reaction (PCR) and sequencing of key oncogenic genes (*meq*, *pp38*, and *vIL-8*). Phylogenetic analysis was conducted using MEGA 12 software to determine the genetic relationships between Tanzanian isolates and MDV strains from Africa and other continents. The results confirm the widespread circulation of MDV in Tanzania, with an overall prevalence of 18.08% across all surveyed zones. Molecular characterization of the *meq*, *pp38*, and *vIL-8* genes revealed high sequence similarity with previously reported MDV strains from Egypt, Nigeria, Israel, and China, with clustering observed in the phylogenetic analysis. Notably, Tanzanian MDV strains exhibited amino acid substitutions associated with increased virulence, particularly in the *meq* gene, which plays a crucial role in MDV-induced tumorigenesis. These findings suggest that MDV strains in Tanzania have undergone genetic changes that could potentially affect vaccine efficacy. Therefore, this study provides valuable information for vaccine manufacturers, poultry farmers, and policymakers in Tanzania, enabling informed decisions when selecting vaccines for MD control.

## 1. Introduction

The poultry industry plays a crucial role in human livelihoods and national economies, leading the growth of livestock production in both developing and developed countries. The demand for poultry and poultry products continues to rise, driven by the growing global population, which is projected to reach 9.8 billion by 2050—potentially doubling the demand for poultry and its products [1]. As a result, the poultry sector has undergone significant transformations. According to the Tanzania Livestock Sector Analysis (2016–2032), Tanzania’s chicken population is estimated to be 83.28 million, consisting of 38.77 million local chickens and 44.51 million exotic chickens (layers and broilers) [2]. Poultry contributes about 16% to Tanzania’s livestock Gross Domestic Product (GDP) and 1% to the national GDP [3,4,5]. Globally, poultry consumption has been increasing due to economic and dietary shifts. In lower-income developing countries, poultry is preferred due to its lower price compared to other meats. Meanwhile, in high-income countries, rising health consciousness and the convenience of white meats have contributed to its growing popularity [6]. Consequently, poultry meat is expected to account for 41% of all protein from meat sources by 2030. Additionally, protein availability from various meat sources is projected to grow by 2030, with poultry leading at 17.8%, followed by sheep meat (15.7%), pork (13.1%), and beef (5.9%) [6]. These trends highlight the critical role of poultry in meeting global protein needs and sustaining the livestock sector’s economic growth.

Beyond economic contributions, poultry farming supports livelihoods through employment, socio-cultural practices, and food security. The rising demand for poultry products has created strong market incentives to improve production efficiency. However, disease outbreaks remain a major constraint, affecting productivity and profitability. In environments where poultry has co-evolved with various challenges, including disease pressure, effective disease control strategies, such as biosecurity measures and vaccination programs, are crucial for sustaining productivity.

Marek’s Disease (MD) is a highly contagious and economically significant viral disease that primarily affects chickens but can also infect other poultry species, including turkeys, pheasants, quail, and gamefowl [7]. The disease is caused by the Marek’s disease virus (MDV), an oncogenic alphaherpesvirus that induces lymphoproliferative disorders in infected birds. Clinically, MD is characterized by asymmetric paralysis, respiratory distress, and lymphomatous tumors in various organs, with mortality rates reaching up to 80% in susceptible flocks [8]. MDV pathogenesis progresses through four overlapping phases: (i) the early cytolytic phase, marked by the initial amplification of the virus in the infected host; (ii) the latent phase, during which latency is primarily established in CD4+ T cells; (iii) the late cytolytic phase; and (iv) the transformation phase, characterized by rapid lymphoma development and the preferential dissemination of tumors to visceral organs and skeletal muscles [9].

MDV exists in three serotypes: (i) Serotype 1, which includes both virulent (e.g., GA, Md11, and Md5) and attenuated vaccine strains (e.g., Rispens/CVI988); (ii) Serotype 2, comprising non-oncogenic strains isolated from chickens (e.g., SB-1); and (iii) Serotype 3, which includes non-oncogenic turkey herpesviruses (HVT) [10]. Serotype 1 is the most oncogenic, capable of inducing tumors in nearly all unvaccinated and genetically susceptible chickens [11]. Based on pathogenicity, serotype 1 strains are further categorized into four pathotypes: mild (mMDV), virulent (vMDV), very virulent (vvMDV), and very virulent plus (vv+MDV) [12].

The MDV genome consists of a double-stranded DNA molecule of approximately 175–180 kb, organized into unique long (UL) and unique short (US) regions, each flanked by terminal and internal repeat sequences [10,13]. Several viral genes contribute to MDV oncogenicity and immune evasion, including the Marek’s EcoRI-Q-encoded protein (*Meq*), viral interleukin-8 (*vIL-8*), latency-associated transcript (LAT), MDV-encoded microRNAs (miRNAs), Repeat long open reading frame 4 (RLORF4), RLORF5a, phosphoprotein 14 (pp14), phosphoprotein 38 (*pp38*), virus-encoded telomerase RNA (vTR), and viral telomeric repeats (TMRs) [9,11,14]. The *Meq* gene comprises a proline/glutamine (Pro/Gln) region, a basic region (BR), and a leucine zipper (ZIP) at the N-terminus, along with a transactivation domain at the C-terminus that contains proline-rich regions, including PPPP motifs [15,16]. The *Meq* gene has four isoforms: the standard isoform, consisting of 339 amino acids (aa); the long *Meq* (L-Meq) isoform, consisting of 398 aa; the short *Meq* (S-Meq) isoform, consisting of 298 aa; the very short *Meq* (VS-Meq) isoform, consisting of 275 aa [16,17]. This diversity in *Meq* arises from variations in the copy number of the PRR region: L-Meq and the standard *Meq* contain 9 and 6 copies of PRR, respectively, while S-Meq and VS-Meq have 4 and 2 copies, respectively [18]. The pp38 phosphoprotein (*pp38*), highly expressed during the lytic phase of MDV infection, plays a crucial role in viral replication and immune modulation, aiding in the transformation of T lymphocytes [19,20,21,22]. Viral interleukin-8 (*vIL-8*) is a secreted CXC chemokine that facilitates the recruitment of MDV target cells, playing a crucial role in MDV pathogenesis and tumorigenesis [9,23,24,25].

Transmission of MDV occurs through the inhalation of infectious virions shed in feather dander [10]. While MD control primarily relies on vaccination, recent reports suggest an increase in vaccine failures [26,27,28]. The emergence of more virulent MDV strains has raised concerns about the continued efficacy of existing vaccines, particularly those based on HVT and CVI988/Rispens [26,29]. Understanding the circulating MDV strains within a specific region is critical for optimizing vaccine strategies and mitigating disease outbreaks.

In Tanzania, Marek’s disease remains a significant challenge for poultry farmers. Previous studies conducted in Morogoro and Dar es Salaam reported high MD prevalence rates of 61.5% and 74.5%, respectively, based on clinical, pathological, and histopathological assessments [30,31]. A five-year diagnostic survey conducted at the Tanzania Veterinary Laboratory Agency (TVLA) in Dar es Salaam (2012–2016) confirmed the endemic nature of MD in the country [30]. However, despite the widespread occurrence of the disease, no molecular characterization of circulating MDV strains has been conducted in Tanzania. This knowledge gap raises concerns about whether the vaccine strains in use (HVT and CVI988) are effective against the field strains circulating in the country.

Between 2010 and 2016, 23 lawsuits were filed by Tanzanian poultry farmers seeking compensation for losses attributed to MD outbreaks, alleging that hatchery owners failed to vaccinate day-old chicks. The validity of these claims remains unverified, necessitating further investigation. Multiple factors could contribute to MD outbreaks in vaccinated flocks, including improper vaccine storage, mismatched vaccine strains, and viral evolution leading to increased virulence [26,32]. However, due to the lack of data on circulating MDV strains in Tanzania, these assumptions remain speculative. This study aimed to detect and characterize the Marek’s disease virus strains circulating in Tanzania using molecular techniques. The sequenced viral genomes were compared to the vaccine strains currently in use (HVT and CVI988) to assess vaccine efficacy. The findings from this study will help guide effective vaccine selection and disease control strategies to mitigate MD-related losses in Tanzania’s poultry industry.

## 2. Materials and Methods

### 2.1. Study Sites

This study was conducted in six livestock representative zones of Tanzania, namely Central (Dodoma), Eastern (Dar es Salaam), Southern (Mtwara), Southern Highlands (Iringa), Lake, and Northern Zone (Mwanza). The selection of study sites based on these zones was preferred due to the following reasons: (i) The potential to detect all MDV strains circulating in the country, (ii) The presence of the Tanzania Veterinary Laboratory Agency (TVLA), which is responsible for investigating livestock diseases in the country, (iii) The availability of diverse poultry production systems, whose operators often bring diseased chickens for investigation, and (iv) The expertise of poultry disease specialists at the TVLAs, who assisted in sample collection.

### 2.2. Study Design

A cross-sectional study was conducted at the selected sites to determine the prevalence and circulating strains of Marek’s disease virus (MDV) in Tanzania. This was aimed at improving the control of Marek’s disease by facilitating the proper selection of vaccines that match the circulating MDV strains.

### 2.3. Samples and Sampling Approach

The study involved the collection of samples from healthy chickens (feather tips) and diseased chickens (kidneys, spleen, liver, and feather tips) from local, layer, broiler, and crossbred breeds. The samples were preserved in RNAlater and stored at −20 °C. During transport, the samples were kept in cool boxes to maintain their integrity. The herpesvirus of turkey vaccine strain currently in use was purchased from vendors and utilized as a positive control.

Computation of the sample size for this study was based on the sample size formula developed by [33].

$$n=\frac{Z^{2}\cdot p\cdot q}{d^{2}}$$

where *n* = sample size, *Z* standard normal deviate = 1.96, *p* = estimated prevalence (50%), and *q* = (1 − *p*), *d* = precision or desired marginal error (5%).

A farm was considered the unit of analysis. Since no prior study had reported the prevalence of Marek’s Disease (MD) in the country, an expected farm-level prevalence of 50% was assumed. Using the formula, a sample size of 384 was calculated. To increase precision, the sample size was doubled, resulting in a final sample size of 769.

Based on the estimated distribution of layer chicken farms across the country, the sample size was allocated across the zones in a ratio of 1:1:1:2:2:3, as shown in Table 1. Within each zone, the allocated sample size was selected randomly. From each farm, a single chicken was sampled to represent the farm.

### 2.4. Sample Processing and DNA Extraction

Tissue samples were homogenized in buffered phosphate saline using a sterile mortar and pestle. The homogenates were collected and stored in cryovials at −80 °C until use. Total genomic DNA was extracted from the samples and the vaccine using the Accu-Prep Genomic DNA Extraction Kit (Bioneer, Daejeon, Republic of Korea) following the manufacturer’s instructions. Sample processing was conducted in a Biosafety Cabinet, while DNA extraction was performed in a Laminar Air Flow. For DNA elution, 50 µL of EA buffer was used. The concentration and quality of the DNA were checked using a NanoDrop Spectrophotometer, and the DNA was stored at −80 °C until use.

### 2.5. Molecular Screening for MDV Field Strain

The extracted DNA from the samples was screened for the MDV field strain using MdCv-F: 5′-GTGATGGGAAGGCGATAGAA-3′ and MdCv-R: 5′-TCCGCATATGTTCCTCCTTC-3′ primers, which were designed in previous studies [34] to target a 225 bp region of the MDV *pp38* gene. The Q5^®^ High-Fidelity DNA Polymerase Kit (New England BioLabs Inc., Ipswich, MA, USA) was used to amplify the target gene. A total of 25 µL of the reaction mixture contained 1× of 5× Q5 reaction buffer, 200 µM dNTPs, 0.5 µM of each forward and reverse primer, 0.02 U/µL of Q5 High-Fidelity DNA Polymerase, and nuclease-free water to achieve the final volume.

The thermocycling conditions for PCR amplification of the target gene were as follows: 98 °C for 30 s (initial denaturation), 35 cycles at 98 °C for 10 s (denaturation), 65 °C for 30 s (annealing), 72 °C for 30 s (extension), followed by a final elongation at 72 °C for 2 min. The PCR products were stained with GreenStar™ Nucleic Acid Staining Solution (Bioneer, Republic of Korea) and separated by electrophoresis through a 1.5% agarose gel. The gel was visualized using UV transilluminators to confirm the specificity of the bands.

### 2.6. PCR Amplification of MDV Oncogenic Genes

Amplification of MDV oncogenic genes targeted the *meq*, *pp38*, and *vIL-8* genes, which are highly associated with viral oncogenicity and pathogenicity [19]. The primers for amplification were adopted from previous studies [29], as shown in Table 2. The amplification conditions and gel electrophoresis for *pp38*, *vIL-8*, and *meq* were as described in the previous section, except for the annealing temperatures, which were 70 °C, 64 °C, and 69 °C, respectively.

### 2.7. Sequencing of the Gene Amplicons and Sequence Analysis

The amplified PCR products of the different genes were sequenced using Sanger sequencing technology in both directions with forward and reverse primers on a commercial basis by Macrogen Company (Amsterdam, The Netherlands). BigDye Terminator v3.1 (Applied Biosystems, Thermo Fisher Scientific, Foster City, CA, USA) and a Genetic Analyzer were used for sequencing. The consensus sequences were generated using BioEdit software (version 7.7.1) and were blasted against sequences in GenBank to determine their identity. Sequence alignment was performed using CLUSTAL W, built into Molecular Evolutionary Genetics Analysis (MEGA) 12 software [35]. Vaccine matching was performed using in silico methods, where sequences from the vaccine and field strains of MDV were compared by aligning the sequences with reference strains and performing phylogenetic analysis in MEGA 12 software. Both nucleotide and deduced amino acid sequences were compared, and residue differences were identified. Phylogenetic trees were generated using the Maximum Likelihood method in MEGA 12 software [35], utilizing up to 7 parallel computing threads. The initial tree for the heuristic search was selected by choosing the tree with the superior log-likelihood between a Neighbor-Joining (NJ) tree [36] and a Maximum Parsimony (MP) tree. The NJ tree was generated using a matrix of pairwise distances computed using the Jones–Taylor–Thornton (1992) model (+Freq) [37]. The evolutionary rate differences among sites were modeled using a discrete Gamma distribution across 5 categories (*+G*, parameter = 200.0000). The analytical procedure encompassed 32 amino acid sequences with 399 positions in the final dataset.

### 2.8. Ethical Clearance

Ethical approval was granted by the Research and Publication Committee of Sokoine University of Agriculture, Morogoro, Tanzania (SUA/RPGS/R/120) on 30 September 2021.

## 3. Results

### 3.1. Screening of MDV Genome by PCR

**Figure 1:**
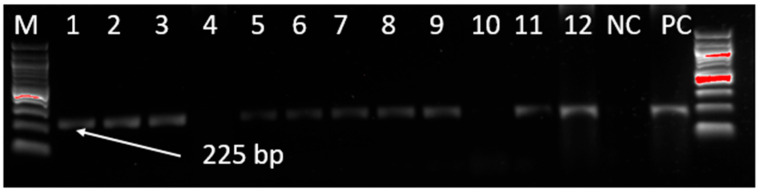
Gel electrophoresis of PCR amplification of the *pp38* gene from MDV field isolates. M denotes the DNA marker (100 bp DNA ladder), 1–12 represent test samples, NC denotes the negative control, and PC denotes the positive control.

The extracted MDV DNA from the samples was screened for the MDV field strain by targeting a 225 bp region of the MDV *pp38* gene. A total of 132 samples producing MDV-specific PCR products of 225 bp were detected out of 784 samples tested (16.84%) collected from Mwanza, Dar es Salaam, Dodoma, Arusha, Iringa, and Mtwara regions (Figure 1).

Samples collected and tested included feather tips and internal organs (liver, kidney, and spleen) from both live and diseased chickens, respectively. The PCR-positive samples from the surveillance zones are shown in Table 3.

### 3.2. PCR Amplification and Sequence Analysis of Meq, pp38, and vIL-8 Genes

All positive samples identified through PCR screening were subjected to amplification of Marek’s disease virus (MDV) oncogenic genes, specifically targeting phosphoprotein 38 (*pp38*), viral interleukin-8 (*vIL-8*), and *meq*. Using gene-specific primers (Table 2), the extracted viral DNA revealed distinct amplification products of approximately 870 bp for *pp38* (S-1 to S-9), 887 bp for *vIL-8* (S-10, S-12, and S-13), and 1026 bp for *meq* (S-15, S-16, and S-18) (Table 4). However, due to a low viral load in some samples, amplification and sequencing of the oncogenic genes were unsuccessful. The generated nucleotide sequences of *meq*, *pp38,* and *vIL-8* were analyzed and submitted to the NCBI GenBank database, and accession numbers were assigned for the isolates (Table 4).

The comparison of nucleotide sequence identity for the *meq* gene detected in this study with reference strains from the NCBI GenBank revealed highly significant alignments (98–100% identity) at 100% query coverage (Table 4). The closest matches were to strains from Hungary (MF431493, collected in 2000), Israel (OQ926512, collected in 2016), Nigeria (OR592064, collected in 2016; MT561535, collected in 2017), Turkey (MW219793, collected in 2016), China (OP887026, collected in 2018), and Iran (MW846296, collected in 2021). The translated amino acid length of the *meq* gene ranged from 212 to 330 amino acids, with mutations observed at positions S71A, D80Y, A88T, Q93R, T139A, P176A, T180A, and P277A when compared to vaccine strains (Table 5). Amino acid substitutions at positions 77, 119, 142 (specific to Chinese vaccine strains), 153, 337, and 386 were consistent with vaccine strains. Amino acid sequence identity with other sequences in the NCBI GenBank, with query coverage ranging from 99% to 100%, varied between 97.19% and 100%. The highest alignments (99% to 100%) were observed with sequences from Egypt (ANF29602, collected in 2013; AXG72667, collected in 2016; QWC49089, collected in 2020), Israel (WOZ50577, collected in 2016), Nigeria (QYL01203, collected in 2017), and Iran (UVJ69088, collected in 2019).

The nucleotide sequence identity of the *pp38* gene detected in this study was compared with reference strains from the NCBI GenBank, revealing a similarity range of 99.66% to 100% at 100% query coverage (Table 5). The nucleotide sequences with the highest significant alignments (100% identity) originated from the Netherlands (PP032833, collected in 1972), Hungary (MF431495, collected in 1970, and MF431493, collected in 2000), China (MW531728, collected in 2008; MG518371, collected in 2011; and KU744561, collected in 2014), Egypt (LC363508, collected in 2015), Tunisia (MN128713, collected in 2016), Thailand (ON931303, collected in 2016), Nigeria (MT561536, collected in 2017), Turkey (MN956507, collected in 2019), and Pakistan (OQ858618, collected in 2021). The translated amino acid sequence of the pp38 gene was 290 residues in length, with observed mutations at position R107Q in samples S-4 to S-6 and S-9. Amino acid sequences showing the highest significant alignments (99.66–100% identity) at 100% query coverage were obtained from China (UOW65371, collected in 2009, and AEC11824, collected in 2010), Egypt (BBC44060, collected in 2015), Thailand (UXN86719, collected in 2016), and Nigeria (QYL01204, collected in 2017).

The nucleotide sequence identity of the *vIL-8* gene detected in this study, compared with reference strains from the NCBI GenBank, ranged from 99.02% to 100% at 100% query coverage (Table 5). The highest significant alignments (100% identity) were observed with sequences from the Netherlands (PP032835, collected in 1972), China (OP887017, collected in 2018), Germany (MT79763, collected in 2019), Pakistan (OQ858618, collected in 2021), and Japan (LC849255, collected in 2022).

The translated vIL-8 gene sequence was 102 amino acids long for all three sequenced DNA products. Amino acid sequences from this study that showed the highest significant alignments (99–100% identity) with reference strains were from the USA (AF147806 and ABG22891) and China (UOW61057). Additionally, amino acid substitutions were observed in sample S-13, with S51P and R64G mutations.

### 3.3. Pairwise Distance and Phylogenetic Analysis of meq Gene

Pairwise comparisons of nucleotide sequences for synonymous and non-synonymous substitution (dN/dS) ratios among three *meq* gene sequences of this study (PV082624, PV082625, and PV082626) were conducted using the codon-based Z-test of selection in MEGA 12 software. These dN/dS ratios were used to infer the type of selection acting on the protein-coding regions. Sequence PV082624 showed a dN/dS ratio of −1.00 with PV082625 and −0.41 with PV082626, indicating purifying selection and suggesting evolutionary pressure to maintain protein function. Sequence PV082625 had a dN/dS of 1.00 with PV082624 and −0.41 with PV082626. Sequence PV082626 showed a dN/dS of 1.00 with both PV082624 and PV082625. Negative dN/dS values (−1.00 and −0.41) reflect purifying selection, where synonymous substitutions are more common, helping preserve protein functionality. In contrast, positive or neutral dN/dS values (around 1.00) suggest relaxed evolutionary constraint or potential positive selection, where non-synonymous changes may be favored. The variation in dN/dS ratios among the sequence pairs indicates differing evolutionary pressures. PV082626 appears to be under less purifying selection compared to the other two sequences. Overall, the average dN/dS ratio was approximately 1, pointing to the possibility of neutral or positive evolution. Further analysis is needed to determine whether any specific sites are under adaptive selection.

The pairwise distance relationships (Supplementary Figure S1) among the amino acid sequences of the *meq* gene (32 amino acid sequences) revealed a minimum pairwise distance close to 0, indicating that some sequences were nearly identical. The maximum pairwise distance varied but reached approximately 0.05, suggesting significant divergence among certain sequences. The average pairwise distance ranged from 0.011 to 0.019, indicating that most sequences were relatively similar (Supplementary Figure S1). Additionally, vaccine strains generally exhibited lower pairwise distances compared to field strains, reflecting their genetic conservation. In contrast, virulent MDV (vMDV) and very virulent MDV (vvMDV) strains had higher maximum distances, signifying greater evolutionary divergence. Notably, the Tanzanian isolates from this study displayed low mean distances (~0.011), suggesting they are closely related.

**Figure 2:**
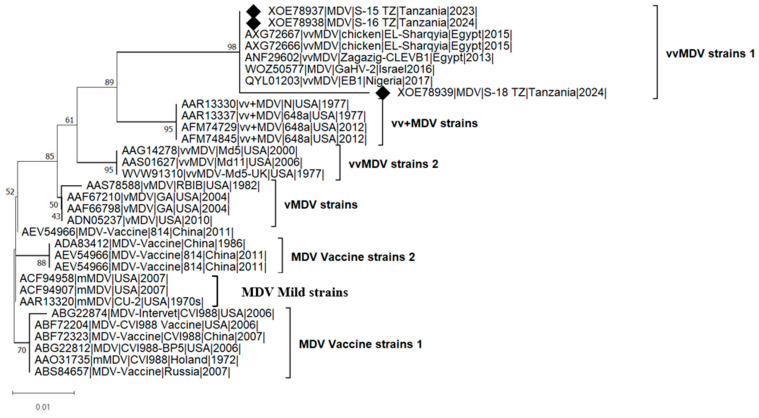
Adaptive bootstrap phylogenetic analysis of the MDV *meq* gene. The phylogeny was inferred using the Maximum Likelihood method and Jones-Taylor-Thornton (1992) model (+Freq) [37] of amino acid substitutions, and the tree with the highest log likelihood (−1225.61) is shown. The percentage of replicate trees in which the associated taxa clustered together, where the number of replicates (109) was determined adaptively [35], is shown next to the branches. The phylogenetic tree was rooted on the branch representing the mild or vaccine MDV strains. The analyses were conducted in MEGA12 [35], utilizing up to 7 parallel computing threads. The symbol “♦” indicates isolates from this study, “v” for virulent, “vv” for very virulent, and “vv+” for very virulent plus strains.

The phylogenetic analysis (Figure 2) of *meq* amino acid sequences was conducted using three isolates from this study, seven sequences from vaccine strains, and 22 additional sequences from previous studies to investigate their genetic relationships. The analysis grouped the isolates into four major clades based on pathotypes: mild MDV (mMDV), which clustered with vaccine strains; virulent MDV (vMDV); very virulent MDV (vvMDV); very virulent plus MDV (vv+MDV). The phylogenetic grouping of MDV strains based on pathotypes is crucial for understanding viral evolution and the progressive increase in its oncogenic potential (virulence) and vaccine resistance. The virulence levels of the MDV strains used in phylogenetic reconstruction were determined through prior experimental pathotyping (infection studies), which assessed virulence based on mortality, tumor formation, and immunosuppression. Vaccine and mild strains formed a distinct clade, separate from virulent, very virulent, and very virulent plus strains, highlighting their evolutionary divergence from field strains over time. The clustering of African strains (Tanzania, Nigeria, and Egypt) suggests independent evolution or regional transmission patterns. However, the grouping of African strains with those from Israel indicates potential intercontinental MDV evolution.

## 4. Discussion

The diagnosis of Marek’s Disease (MD) in Tanzania has traditionally relied on clinical signs, gross pathology, and histopathology without confirmation through virus isolation or molecular techniques. This study provides an in-depth molecular characterization of Marek’s Disease Virus (MDV) strains circulating in Tanzania, offering critical insights into their genetic diversity and potential implications for disease control strategies. The detection of MDV-positive samples in all six disease surveillance zones suggests that MD remains a significant threat to poultry production in Tanzania. The overall prevalence of 18.08% indicates widespread viral circulation, underscoring the need for effective disease management measures. Furthermore, mutations identified in the MDV oncogenic genes present challenges for existing control strategies.

The genetic analysis of the *meq*, *pp38*, and *vIL-8* oncogenic genes revealed high sequence similarity with strains previously reported in Egypt, Nigeria, Israel, and China. This study identified the short *Meq* (S-Meq) isoform of 330 amino acids with notable mutations at specific positions, which may influence the virus’s oncogenic potential. The *meq* gene encodes a protein critical for MDV-induced tumorigenesis and has been associated with increased virulence [11,16,38]. The long isoform of Meq (L-Meq), observed in vaccine strains such as CU-2 and CVI988/Rispens, contains an insertion of a 59/60 amino acid segment in the proline-rich regions, forming eight consecutive PPPP sequences, which suppresses *meq* expression [16,39].

A total of 14 amino acid substitutions were identified in the *meq* gene, with six substitutions (77, 119, 142, 153, 337, and 386) matching those in vaccine strains, while eight substitutions (71, 80, 88, 93, 139, 176, 180, and 277) differed from vaccine strains. Notably, the substitutions at positions 71, 80, 88, 93, 139, 176, 180, and 277 were identical to those found in Nigerian isolates (WYC13993 and QYL01231) and Egyptian isolates (AXG72666, AXG72667, and ANF29602). Sequence alignment of the deduced amino acid sequence of the Meq protein (Table 4) indicates that virulent strains have amino acid substitutions at positions S71A, E77K, and P277A, while very virulent strains exhibited substitutions at S71A, D80Y, A88T, Q93R, T139A, P176A, T180A, and P277A. Very virulent plus strains display substitutions at S71A, E77K, C119R, P153Q, P176A, T180A, and P277A.

Amino acid substitutions at position 71 are present in all virulent pathotypes, while positions 80, 88, 93, and 139 are specific to very virulent strains, and positions 119 and 153 are exclusive to very virulent plus strains. Substitutions at positions 71, 80, 115, 139, 176, and 217 have been associated with very virulent strains causing more than 50% mortality in China [40]. Conversely, substitutions at positions 88, 93, and 139 have been linked to less virulent strains and may contribute to reduced virulence [40,41]. Comparative amino acid sequence analysis of *meq* genes across the three pathotypes of virulent MDV (vMDV, vvMDV, and vv+MDV) has indicated point mutations in the proline-rich repeats (the four consecutive PPPP sequences) at positions 153, 176, 217, and P277A [19]. Disruption of the PPPP sequences, where the second proline residue is substituted with another amino acid, has been associated with highly virulent MDV strains [42].

The *pp38* gene, which plays a key role in early cytolytic infection and lymphoid cell transformation [22,43], exhibited mutations at positions 107 and 115 in some Tanzanian isolates. These variations may influence viral replication and pathogenicity. Similarly, the *vIL-8* gene, which is involved in immune evasion and viral reactivation [26,44], displayed genetic differences compared to reference strains. Such mutations could potentially impact viral transmission dynamics and host immune responses.

The pairwise dN/dS analysis of the *meq* gene sequences from Tanzanian Marek’s disease virus (MDV) isolates revealed evidence of both purifying and neutral/positive selection. Negative dN/dS values suggest purifying selection acting to conserve critical viral functions, a trend previously observed in MDV evolution [45,46,47]. Conversely, values around 1.00 indicate relaxed selective pressure or potential adaptive evolution, possibly driven by host immune responses or vaccine-induced selection [48]. Pairwise distance analysis of *meq* amino acid sequences showed low mean distances (~0.011) among Tanzanian isolates, suggesting limited genetic drift and close relatedness [49]. In contrast, higher divergence (up to ~0.058) among virulent and very virulent strains supports the global pattern of MDV evolution under vaccine and host immune pressure [50,51]. These findings align with previous studies showing lower divergence in vaccine strains and greater variation in very virulent field strains [52].

Phylogenetic analysis revealed further that the Tanzanian MDV isolates cluster within the very virulent MDV (vvMDV) clade, clearly distinct from vaccine and mild strains, indicating their potential role in field outbreaks. This divergence supports previous findings that field strains are evolving separately from vaccine lineages [53,54]. The close genetic relationship between Tanzanian isolates and vvMDV strains from other African countries and Israel suggests possible regional transmission or intercontinental viral movement, consistent with global phylogeographic trends [47]. These findings raise concerns about the ongoing efficacy of existing vaccines, as the presence of mutations in oncogenic genes points to ongoing viral evolution under selection pressure, likely driven by widespread vaccine use. Similar evolutionary patterns have been documented in other regions, where vaccination has been linked to the emergence of more virulent MDV strains [7,48,55].

Herpesvirus of Turkey (HVT) and CVI988/Rispens are widely used vaccines for controlling MD in Tanzania. However, previous histopathological studies [30,31] and findings from this study indicate that MD remains endemic in Tanzanian poultry populations. This persistence is likely due to factors such as widespread MDV presence, limited vaccination coverage, poor biosecurity measures, and environmental conditions such as high temperatures and humidity, which influence viral stability and transmission. Previous studies have shown that the protective efficacy of four commercial MD vaccines (SDCW01, CVI988, HVT, and CVI988+HVT) was relatively low, with protection indices of 46.2%, 38.5%, 50%, and 28%, respectively [56]. Additionally, chickens vaccinated with monovalent CVI988 or HVT still developed tumors, with cumulative incidences of 7.7% and 11.5%, respectively. Vaccine failures have been reported in regions with highly virulent MDV strains, leading to outbreaks even in HVT and HVT+SB-1 vaccinated flocks, with chicken mortality rates exceeding 60% due to selection pressure imposed by HVT vaccination [26,40]. The low protection indices may be attributed to mutations in MDV isolates, resulting in the emergence of highly virulent strains [56,57]. It is normally expected that bivalent vaccines, such as CVI988+HVT, provide enhanced protection due to the combination of two different vaccine strains. However, in this case, it shows the lowest protection index (28%). The lower protection index of CVI988+HVT is likely due to: (i) viral interference between CVI988 and HVT during replication in the host cells, leading to reduced replication and immunogenicity of either or both strains; (ii) incompatibility or mismatch with circulating local MDV field strains that are genetically distant from HVT and CVI988; and (iii) possibly host-related factors. In contrast, SDCW01, possibly a local recombinant vaccine, demonstrated higher protection (46.2%), emphasizing the importance of genotype-matched vaccines for controlling vv+MDV strains.

This study has identified and characterized very virulent MDV strains circulating in various surveillance zones in Tanzania, which may contribute to vaccine failures. Given the observed genetic diversity, continuous molecular surveillance of MDV is essential for monitoring viral evolution and assessing vaccine effectiveness. Future research should focus on pathogenicity trials to determine the virulence of detected strains and their impact on poultry health. Additionally, vaccine manufacturers and policymakers should consider these findings when selecting appropriate vaccines for MD control in Tanzania.

The study also highlights the limitations of current vaccination strategies in Tanzania. The reliance on HVT and CVI988/Rispens vaccines may not be sufficient for long-term protection against evolving MDV strains. Given the low protection indices of commercial vaccines and reports of vaccine failures in regions with highly virulent strains, alternative or combination vaccine strategies should be explored. For example, the vaccination of chickens with an attenuated *meq* and a cytolytic replication-related gene, *pp38* deletion mutant virus, has shown superior protection against highly virulent viruses compared with the commercial vaccine strain CVI988/Rispens [58]. Furthermore, inadequate vaccination coverage, particularly among small-scale and backyard poultry farmers, contributes to the continued spread of MDV. Strengthening biosecurity measures, enhancing vaccination protocols, and developing region-specific vaccines should be considered to mitigate MD outbreaks in Tanzania.

## 5. Conclusions

This study provides the first molecular characterization of circulating MDV strains in Tanzania, revealing the presence of highly virulent variants with significant genetic divergence from vaccine strains. The findings underscore the importance of continuous molecular surveillance to monitor viral evolution and assess vaccine effectiveness. Given the observed genetic diversity, vaccine manufacturers and policymakers should consider revising vaccination strategies to ensure better protection against emerging MDV strains. Future research should focus on pathogenicity trials to determine the virulence of the detected strains and evaluate their impact on poultry health. Implementing targeted vaccination programs, combined with enhanced biosecurity measures, will be crucial in controlling MD and sustaining poultry production in Tanzania.

## Figures and Tables

**Table 1:** The distribution of the Sample size across zones.

Zone/Region	Ratio	Multiplier	Sample Size
Southern/Mtwara	1	0.1	77
Central/Dodoma	1	0.1	77
Southern Highland/Iringa	1	0.1	77
Northern/Arusha	2	0.2	154
Lake/Mwanza	2	0.2	154
Eastern/Dar	3	0.3	230
Total sample size (chickens)			769

**Table 2:** Primers for amplification of oncogenic genes and their amplicon size.

Gene	Forward Primer	Reverse Primer	Amplicon Size
*meq*	5′-GGCACGGTACAGGTGTAAAGAG-3′	5′-GCATAGACGATGTGCTGCTGAG-3′	1081 bp
*pp38*	5′-TCATCTTCAACCCACAGCCATCC-3′	5′-TCGCTTAATCTCCGCCTCCAAC-3′	1006 bp
*vIL-8*	5′-GAGACCCAATAACAGGGAAATC-3′	5′-TAGACCGTATCCCTGCTCCATC-3′	887 bp

**Table 3:** Total number of feather tips and internal organs tested positive for MDV by PCR in the study zones/regions.

Zone/Region	Total Number of Samples Collected	PCR Positive by Sample Type		PCR Positive Samples by Zone/Region (Prevalence)
		Feather Tips (Live Chickens)	Internal ORGANS (DISEASED)	
Lake/Mwanza	154	27 (n = 125)	25 (n = 29)	52 (33.77%)
Eastern/Dar es Salaam	230	8 (n = 91)	22 (n = 139)	30 (13.04%)
Central/Dodoma	77	0 (n = 31)	4 (n = 46)	4 (5.19)
Northern/Arusha	154	7 (n = 129)	3 (n = 25)	10 (6.49%)
Southern Highland/Iringa	77	10 (n = 67)	3 (n = 10)	13 (16.88%)
Southern/Mtwara	77	15 (n = 55)	8 (n = 22)	23 (29.87%)
Total	769	67 (n = 498)	65 (n = 271)	132 (18.08%)

**Table 4:** Lengths of the sequenced PCR products, translated amino acid sequences, their identities, and the corresponding accession numbers for the oncogenic genes.

Sample ID	Collection Date	Location	Target Gene	Nucleotide Length (nt)	Nucleotide Identity (%)	Amino Acids Length (aa)	Amino Acid Identity (%)	Accession Number
S-1	7 December 2023	Mwanza	*pp38*	870	99.66–100	290	97.93–100	PV008123
S-2	12 December 2023	Mwanza	*pp38*	870	99.66–100	290	97.93–100	PV008124
S-3	13 March 2024	DSM	*pp38*	870	99.31–99.77	290	99.43–99.77	PV008125
S-4	15 March 2024	DSM	*pp38*	870	99.66–99.89	290	98.28–100	PV008126
S-5	18 November 2024	Dodoma	*pp38*	870	99.54–99.89	290	96.47–100	PV008127
S-6	10 September 2023	Arusha	*pp38*	786	99.62–100	262	98.85–100	PV008128
S-7	18 November 2024	Iringa	*pp38*	870	99.77–100	290	98.28–100	PV008129
S-8	2 November 2023	Mtwara	*pp38*	870	99.77–100	290	91.44–100	PV008130
S-9	5 November 2023	Mtwara	*pp38*	870	99.66–100	290	97.93–100	PV008131
S-10	7 December 2023	Mtwara	*vIL-8*	537	99.26–100	102	95.10–100	PV008132
S-12	13 March 2024	DSM	*vIL-8*	816	99.02–99.88	102	99.02–100	PV008133
S-13	18 November 2024	Dodoma	*vIL-8*	832	98.80–100	102	97.06–100	PV008134
S-15	7 December 2023	Mwanza	*meq*	1026	98.83–99.42	330	98.18–100	PV082624
S-16	15 January 2024	Arusha	*meq*	1026	98.83–99.42	330	98.18–100	PV082625
S-18	18 November 2024	Dodoma	*meq*	654	97.40–98.16	212	92.40–95.75	PV082626

**Table 5:** Sequence alignment of the deduced amino acid sequence of meq protein.

Country	Strain	Type	Code	71	77	80	88	93	119	139	153	176	180	277	337
USA	CVI988-BP5	Vaccine	ABG22812	S	E	D	A	Q	C	T	P	P	T	P	L
USA	CVI988	Vaccine	ABF72204	S	E	D	A	Q	C	T	P	P	T	P	L
China	814	Vaccine	AEV54966	S	E	D	A	Q	C	T	P	P	T	P	L
Holand	CVI988	mMDV	AA031735	S	E	D	A	Q	C	T	P	P	T	P	L
USA	CU-2	mMDV	AAR13320	S	E	D	A	Q	C	T	P	P	T	P	L
USA	RBIB	vMDV	AAS78588	A	K	D	A	Q	C	T	P	P	T	P	L
USA	GA	vMDV	AAF67210	A	K	D	A	Q	C	T	P	P	T	P	L
USA	Md11	vvMDV	AAS01627	A	K	D	A	Q	C	T	P	P	T	A	L
Nigeria	Nigeria	vvMDV	WYC13993	A	E	Y	T	R	C	A	P	A	A	A	L
Nigeria	EB1	vvMDV	QYL01203	A	E	Y	T	R	C	A	P	A	A	A	L
Egypt	EL-Sharqyia	vvMDV	AXG72666	A	E	Y	T	R	C	A	P	A	A	A	L
Egypt	EL-Sharqyia	vvMDV	AXG72667	A	E	Y	T	R	C	A	P	A	A	A	L
Egypt	CLEVB1	vvMDV	ANF29602	A	E	Y	T	R	C	A	P	A	A	A	L
Tanzania	S-15_TZ	vvMDV	XOE78937	A	E	Y	T	R	C	A	P	A	A	A	L
Tanzania	S-16_TZ	vvMDV	XOE78937	A	E	Y	T	R	C	A	P	A	A	A	L
Tanzania	S-18_TZ	vvMDV	XOE78937	A	E	Y	T	R	C	A	P	A	A	A	L
USA	684a	vv+MDV	AFM74845	A	K	D	A	Q	R	T	Q	A	A	A	L
USA	N	vv+MDV	AAR13330	A	K	D	A	Q	R	T	Q	A	A	A	L

## Data Availability

Data Availability Statement: All genetic data sets in this study can be found at https://www.ncbi.nlm.nih.gov/ (accessed on 25 March 2025).

## Supplementary Materials

The following supporting information can be downloaded at: https://www.mdpi.com/article/10.3390/v17050698/s1, Figure S1: The heatmap illustrating the pairwise distance relationships among the amino acid sequences of *meq* gene. Light red areas represent closely related sequences (low distance), dark red areas indicate higher divergence and lighter shades show intermediate levels of similarity. The minimum pairwise distance is close to 0, meaning some sequences are nearly identical. The maximum distance varies but can go up to ~0.05, indicating some sequences have significant divergence. The pairwise distances were computed using MEGA 12 and the heatmap was constructed using ggplot2 package of R software (R 4.4.3).
