# Supplementary material for: Surveillance and Molecular Characterization of Marek’s Disease Virus (MDV) Strains Circulating in Tanzania
Source: Viruses. 2025 May 13;17(5):698. doi: 10.3390/v17050698 (PMC12115803; doi:10.3390/v17050698)
Supplement: Supplementary file 1 [file viruses-17-00698-s001.zip › viruses-3582496-supplementary.pdf]

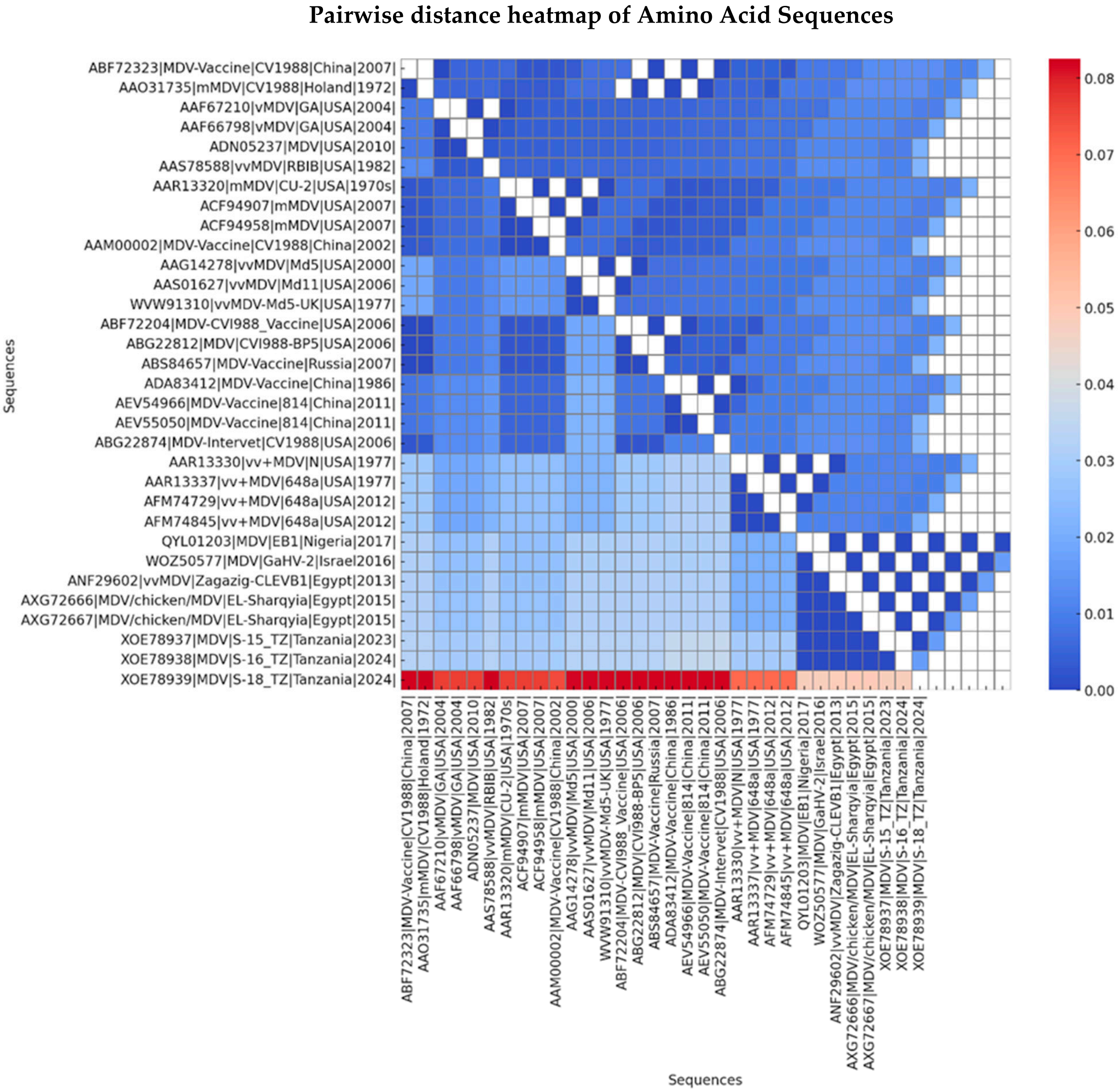

Figure S1. The heatmap illustrating the pairwise distance relationships among the amino acid sequences of *meq* gene. Dark blue areas represent closely related sequences (low distance), dark red areas indicate higher divergence and lighter shades show intermediate levels of similarity. The minimum pairwise distance is close to 0, meaning some sequences are nearly identical. The maximum distance varies but can go up to ~0.08, indicating some sequences have significant divergence.
